# Pathophysiology of Pediatric Traumatic Brain Injury

**DOI:** 10.3389/fneur.2021.696510

**Published:** 2021-07-15

**Authors:** Rebecka O. Serpa, Lindsay Ferguson, Cooper Larson, Julie Bailard, Samantha Cooke, Tiffany Greco, Mayumi L. Prins

**Affiliations:** ^1^Department of Neurosurgery, Brain Injury Research Center, David Geffen School of Medicine, University of California, Los Angeles, Los Angeles, CA, United States; ^2^Steve Tisch BrainSPORT Program, University of California, Los Angeles, Los Angeles, CA, United States

**Keywords:** pediatric, adolescence, traumatic brain injury, metabolism, inflammation, long term outcome, behavior

## Abstract

The national incidence of traumatic brain injury (TBI) exceeds that of any other disease in the pediatric population. In the United States the Centers for Disease Control and Prevention (CDC) reports 697,347 annual TBIs in children ages 0–19 that result in emergency room visits, hospitalization or deaths. There is a bimodal distribution within the pediatric TBI population, with peaks in both toddlers and adolescents. Preclinical TBI research provides evidence for age differences in acute pathophysiology that likely contribute to long-term outcome differences between age groups. This review will examine the timecourse of acute pathophysiological processes during cerebral maturation, including calcium accumulation, glucose metabolism and cerebral blood flow. Consequences of pediatric TBI are complicated by the ongoing maturational changes allowing for substantial plasticity and windows of vulnerabilities. This review will also examine the timecourse of later outcomes after mild, repeat mild and more severe TBI to establish developmental windows of susceptibility and altered maturational trajectories. Research progress for pediatric TBI is critically important to reveal age-associated mechanisms and to determine knowledge gaps for future studies.

## Introduction

Addressing traumatic brain injury (TBI) in the pediatric population is exceptionally complex. The pediatric population is a heterogeneous age group that spans across numerous significant developmental milestones. Epidemiological data from the Centers for Disease Control and Prevention (CDC) shows a bimodal increase in the incidence of TBI within the pediatric population with annual incidence of 315,979 among children between 0 and 4 years of age and 475,876 among adolescents (15–24 years old) (1). The distribution of TBI between the sexes also differs, with males comprising 55.3% of children (age 0–4) and 62.8% of adolescent (age 15–19) TBIs (2). Types of brain injuries between both children and adolescent groups also differ. Toddlers/young children are more likely to sustain a TBI from a fall vs. adolescents who are more likely to suffer injuries from sports, motor-vehicle accidents, and assaults (1, 3). The heterogenous nature of brain maturation compounded by emergence of sex differences and different types of injuries sustained, makes assessment of pathophysiological responses, vulnerabilities, and recovery trajectories very difficult. Adding to this are socioeconomic status, insurance disparities, and racial inequities that can contribute to long-term outcomes and recovery success in both children and adolescent TBI patients. This review will address the known acute and long-term pathophysiological mechanisms for children and adolescent TBI to provide clinical and preclinical timelines to guide future research.

## Acute Pathophysiological Changes

### Glucose and Cerebral Blood Flow

**Figure 1:**
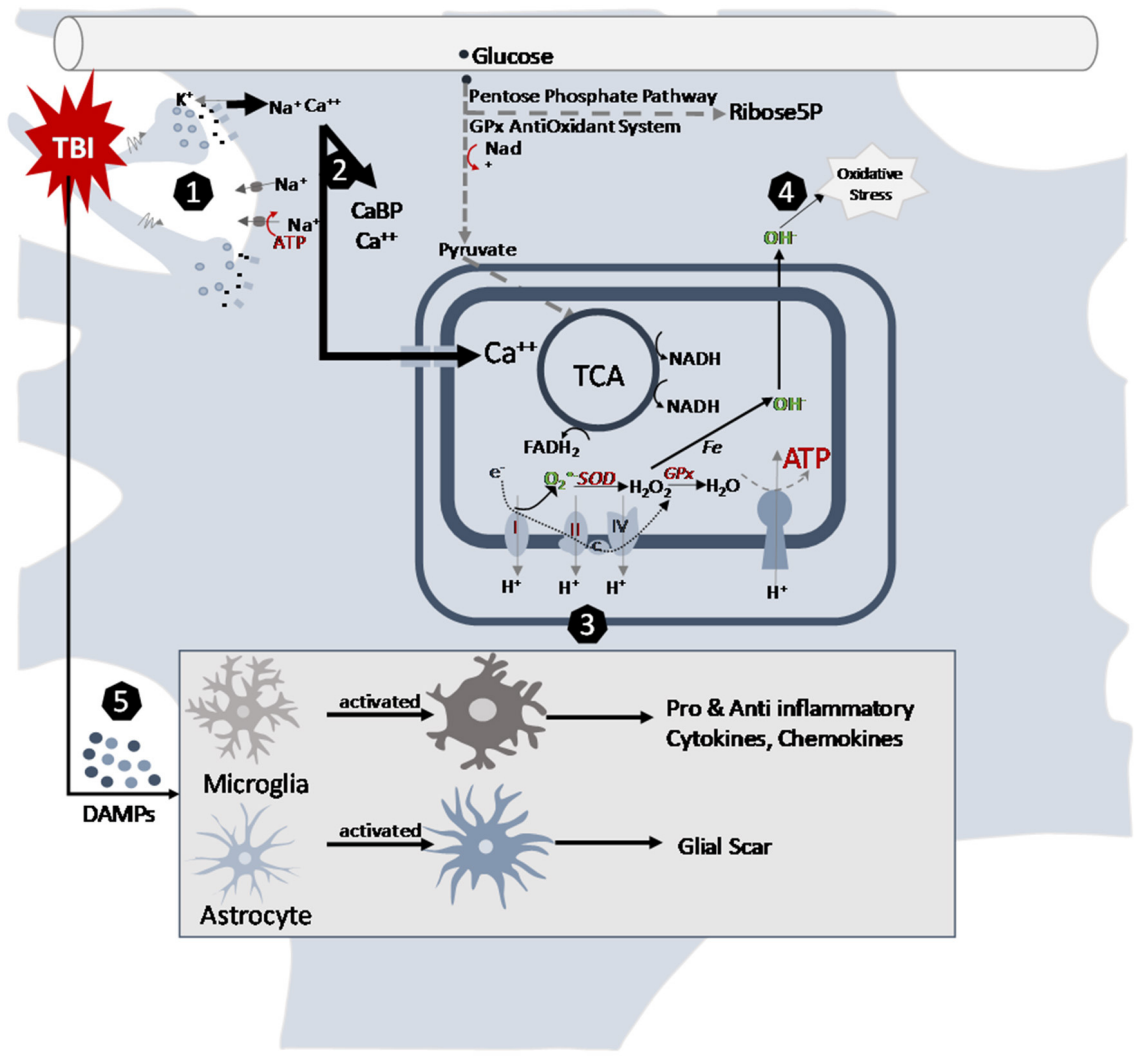
Cellular pathways altered by traumatic brain injury in the juvenile brain. (1) Mechanical movement of the brain tissue causes massive depolarization of neurons. The indiscriminate release of neurotransmitters opens postsynaptic receptors and intermembrane ion concentrations are disrupted as ions flow down their concentration gradients. Na^+^and Ca^++^ accumulates in the cells. Energy demanding ATPase pumps are activated to return ionic homeostasis. (2) Calcium accumulation is managed by calcium binding proteins and sequestration into mitochondria after TBI. (3) Changes in mitochondrial electron transport enzyme activities after TBI contribute to acute decrease ATP production and increase in reactive oxygen species production. The slower maturing anti-oxidant systems contribute to younger age vulnerabilities. (4) Metabolism induced oxygen free radicals form along the electron transport chain. Maturational lower levels of mitochondrial superoxide dismutase (SOD) and antioxidant enzyme glutathione peroxidase (GPx) along with the younger brain's lessened upregulation after TBI limit their capacity for reactive oxygen species management. This contributes to the oxidative stress of the cells. (5) After TBI, neurons release damage-associated molecular patterns (DAMPs), which induce morphological changes in astrocytes and microglia. In their activated states, microglia release cytokines and chemokines and astrocytes can inhibit microglial signaling and generate glial scarring that then contribute to the inflammatory cascades post injury.

**Figure 2:**
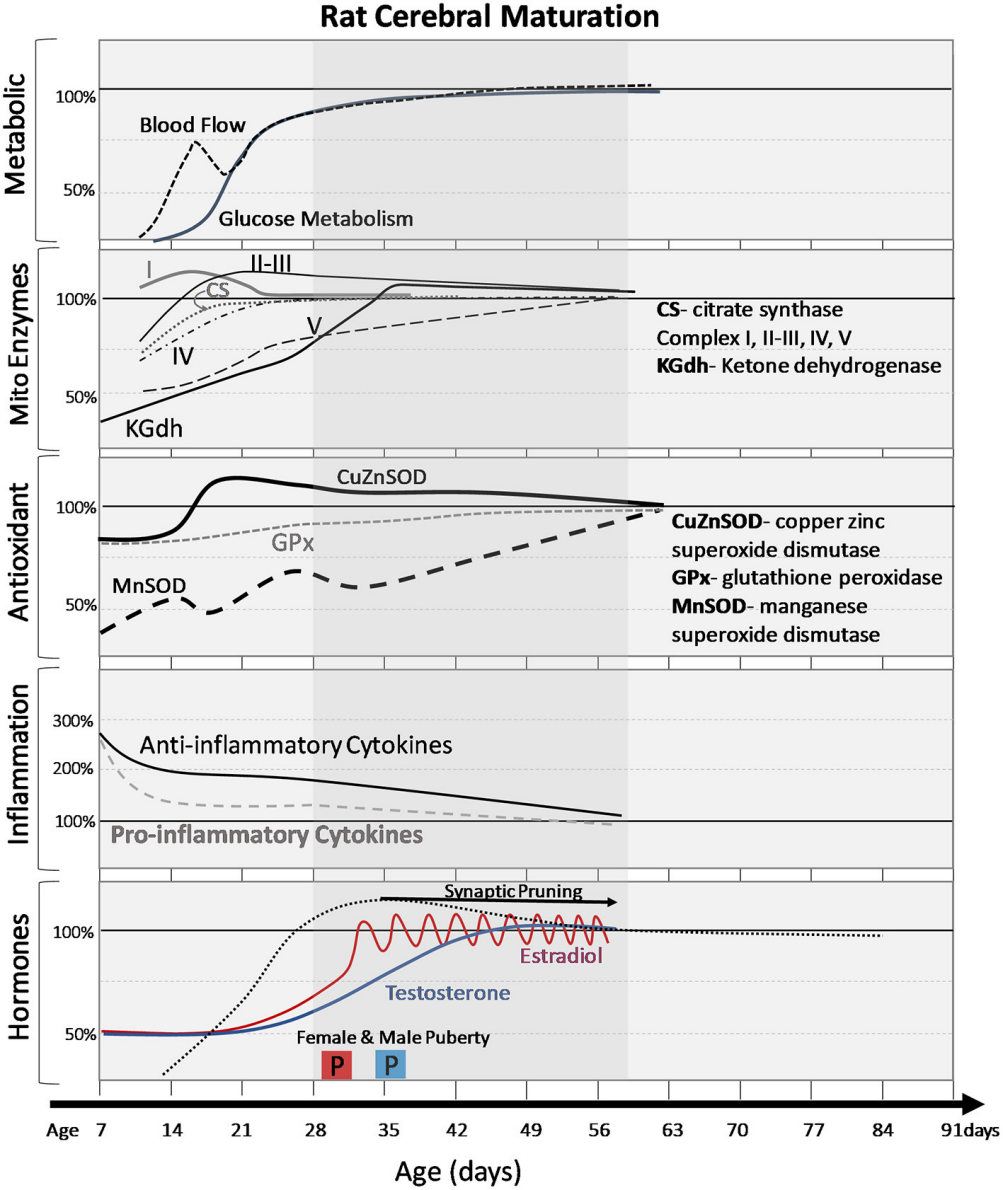
Normal cerebral changes in metabolic markers, mitochondrial enzymes, antioxidants, inflammatory signals, and hormones with postnatal age (days). The shaded age range reflects adolescent time period. Changes are expressed as percentage of adult values. Pro and anti-inflammatory cytokines are averages of multiple cytokines to demonstrate general trends. Onset of puberty (2 boxes labeled “P”) is earlier in females (pink box) than males (blue box) and the differences in timing of hormonal increases and patterns (gradual increase vs. pulsatile changes in females).

There are known developmental changes in the brain's reliance on metabolic substrates, metabolic rates, and cerebral blood flow (CBF). Shortly after birth when nursing begins, the developing brain relies on both ketones and glucose until “weaning,” or reduced milk intake. The normal developmental profile for glucose metabolism and CBF increases slowly toward adulthood in the rat (4, 5) and decreases gradually between 4 and 16 years of age in humans (6, 7) (Figures 1, 2). It is important to understand the normal developmental patterns across age groups to anticipate how TBI may impact maturational profiles.

Research examining changes in glucose metabolism after mild TBI (mTBI) or repeat mild TBI (rTBI) is sparse. Mild weight drop injury in male adult Sprague Dawley rats showed no significant decrease in cerebral metabolic rate of glucose (CMRg) at 12 d, 1, or 3 months post-injury (8). Similar lack of brain glucose metabolic changes was observed in human patients with uncomplicated mTBI and no MRI lesion at 6 months post-injury (9). This evidence suggests that alterations in brain glucose metabolism after mTBI are acutely transient, but not chronic. Research after mTBI in pediatric patients is lacking, but preclinical studies have been conducted during the acute period. A single mild concussive closed head injury in adolescent male rats at postnatal day 35 (P35), resulted in a significant 17% decrease in CMRg at 1 d, which recovered to sham levels by 3 days post-injury (10). When a second injury was introduced 24 h after the first injury, the CMRg decreased by 35% and remained 21% decreased at 3 days post-injury compared to shams. However, if the second injury was introduced 3 days after the first injury, the magnitude of CMRg depression and recovery were similar to the single impact, suggesting a metabolic window of vulnerability. Changes in brain glucose metabolism have been shown to increase in magnitude and duration as TBI severity increases (11). Results from a moderate lateral fluid percussion injury (FPI) in P17, P28, and adult rats also demonstrate that magnitude and duration of CMRg depression increases with age. The 2 days post-injury CMRg depression was 3, 13, and 20% compared to shams in P17, P28, and adults, respectively. In the developing animal metabolic recovery was achieved by 5 days post-injury, while CMRg in adults continued to remain depressed at 14 days post-injury (12, 13).

The methods to measure CMRg reflect brain glucose catalyzes by glycolytic enzyme hexokinase, but not where glucose carbons are utilized after injury. Glucose carbons have several biochemical pathways through which they can be processed. Glycolysis has 13 enzymatic steps before the carbons can enter the mitochondria for energy production. A key rate-limiting enzyme glyceraldehyde phosphate dehydrogenase (GAPDH) is the 5th step in this pathway. GAPDH requires cytosolic nicotinamide adenine dinucleotide (NAD^+^) as a co-factor. Studies measuring cytosolic NAD^+^ levels after TBI in adult and adolescent rats show that there is a significant decrease in NAD^+^ availability at 6–12 h in adults, and 12–24 h in adolescent brains (11). The decreased availability of this co-factor will decrease the ability of GAPDH to process glucose through glycolysis. While glycolysis remains inhibited, metabolism of glucose carbons are upregulated in other pathways. The concomitant upregulation of the pentose phosphate pathway (PPP) allows for production of byproducts necessary for DNA repair and activation of glutathione peroxidase (GPx) for detoxification of oxidative stress. ^13^C-glucose NMR studies in adults after controlled cortical impact (CCI) injury show a 9–13% increase in glucose shunting toward this pathway (14). Similar studies have not yet been done in developing age groups.

### Calcium

Calcium is a critical cellular messenger in healthy neurons, and its intracellular concentration is maintained at a level five times lower than its extracellular concentration. This strong concentration gradient precipitates an acute ionic influx after neuronal injury. Rapid brain movement stretches neurons resulting in mechanoporation and early indiscriminate activation of N-methyl-D-aspartate (NMDA) receptors allowing an influx of sodium and calcium ions into cells from the extracellular space (11). The disruption of ionic homeostasis increases demand for cytosolic ATP thereby increasing the demand for glucose. The movement of calcium following injury can thus influence metabolic dysfunction following TBI.

Regional and age-dependent patterns of calcium accumulation emerge after mild and repetitive mild TBI (rTBI) in a variety of animal models. Calcium-labeled autoradiography was used following mild lateral FPI in P17 and P28 and adult rats, to show acute, diffuse increases in cortical calcium accumulation (15). Recovery to sham levels was achieved in 2–4, 1, and 4 days in P17, P28, and adult rats, respectively. Cortical calcium accumulation in P17 and P28 rats manifested as a peak followed by gradual recovery, whereas calcium levels in adult rats plateaued during these early timepoints (15). A bimodal peak in calcium accumulation was observed in P28 and adult rats in the ipsilateral thalamus starting at 4 and 2–4 days post-injury respectively, increasing out to 14 days in both groups (15). Histology in these regions implicate calcium release following cell death for this later thalamic increase in calcium, as opposed to a separate independent insult to the blood-brain barrier (BBB). The P17 group did not show this thalamic increase pattern, suggesting that post-traumatic calcium accumulation is age-dependent (Figure 1). Immunohistochemical analysis of calcium-activated cytosolic calpain proteases in P11 and P17 rats exposed to closed skull injury support this age-dependent dual timeline of calcium accumulation (16). Calpain activation in the cortex increased within 24 h after injury and waned by the third day in P11 rats (16). While P17 rats similarly did not display a change in calpain activation in the thalamus, P11 rats exhibited a delayed increase in calpain activation 3 days after injury (16). Even after recovery, mild pediatric TBI may continue to influence calcium homeostasis.

Calcium accumulation is generally managed by calcium binding proteins or sequestration in mitochondrial and endoplasmic reticulum within the cell. Calcium binding proteins (CaBP, including S100, calmodulin, calbindin, and parvalbumin) have been observed to attenuate the excitotoxic calcium influx by binding calcium within the cytosol. However, developing brains have lower levels of CaBP expression in early postnatal development, potentially reducing their ability to buffer excitotoxic calcium influx, or increasing their susceptibility to apoptosis (17, 18). Preclinical studies in rats demonstrate increasing CaBP expression throughout development with patterns varying regionally (18). Calbindin and parvalbumin immunoreactivity in rats increase throughout cortical development reaching adult levels at the end of the third postnatal week (17, 19). While clinical studies have examined S100 in blood or cerebral spinal fluid (CSF) after human TBI (20), there are limited developmental studies addressing expression changes after TBI. A single study using a mechanical drill penetration injury in the developing rat brain did show earlier increases in astrocytic S100 expression in P6 and P14 day rats compared to P30 rats (21). Another study of P28 mice saw that S100B enhanced neuronal cell survival and migration in the granular layer of the hippocampus, but failed to enhance proliferation in the subgranular zone 4 days post-injury (22). It remains unclear how TBI and CaBP maturational changes intersect.

In addition to cytosolic calcium binding proteins, the mitochondria also play a crucial role in maintaining intracellular calcium homeostasis by buffering the acute calcium influx after TBI (23). The mitochondrial calcium uniporter (MCU), alongside voltage-dependent anion channels (VDACs), transport Ca^2+^ from the cytosol to the intermembrane space (24–26). Excessive calcium sequestration in the intermembrane space can promote mitochondrial dysfunction by stimulating the formation of the mitochondrial permeability transition pore (mPTP) (27). Preclinical evidence suggests that brains in different stages of development may have different capacities for mitochondrial calcium sequestration (28). In the presence of ATP, isolated brain mitochondria from P16–18 rats exhibit lower maximal calcium uptake than adults (29). In the absence of ATP, however, the opposite occurs, which may affect energy-deficient, hypoxic conditions after TBI (30).

### Mitochondria

Many facets of mitochondrial function show developmental profiles that are important to understand prior to addressing age related injury responses. Multiple preclinical studies have described the steady increase in oxygen consumption, ADP/Oxygen ratios, and the respiratory control ratio (RCR) with maturation of the developing rat brain (31–33). Non-synaptic mitochondria are located in the axons, soma, and dendrites of neurons, and within non-neuronal cells. Within this population of mitochondria the increase in respiration is mirrored by an increase in the activities of electron transport chain (ETC) complexes I and V to adult levels by P21 (34). Complexes II, III, and IV develop slower, reaching adult levels of activity by P60 (34). Synaptic mitochondria are located within nerve synapses, sites of high energy demand and calcium influx. These do not appear to follow the same pattern of ETC complex activity as their non-synaptic counterparts. Complex V activity was found to be higher over the course of development in synaptic mitochondria compared to non-synaptic mitochondria, whereas Complex I activity was lower in synaptic mitochondria (35). Taken together, these findings suggest that there may be heterogeneity in the response to injury between cell types at different stages of development (Figure 1).

In addition to the enzymes in the ETC, key enzymes involved in the tricarboxylic acid (TCA) cycle develop relatively slowly in the rat brain. The rate limiting enzyme of the TCA cycle, alpha-ketoglutarate dehydrogenase, has a characteristic increase in concentration across nearly all brain regions to adult levels by P30 in the rat (36). While citrate synthase activity increases beginning at P8, reaching adult levels by P21, pyruvate dehydrogenase complex (PDH) activity develops less quickly, increasing by P15, only reaching 40–60% of adult levels by P21 (37). The observed increase in PDH activity at P15 coincides with a decrease in the level of circulating beta-hydroxybutyrate (BHB) from peak levels between P11–15 (38). P10–17 rats also display a greater capacity for cerebral ketone body uptake than adult rats before rates decrease significantly during weaning between P17–21 (39). Studies have shown that while glucose is the primary cerebral energy fuel at all ages and across species (40), ketone bodies account for up to 20% of total energy production in the developing brain (41).

TCA and ETC enzymes are mitochondrial energy producing enzymes, but there are also important enzymes involved in protecting the cells from a byproduct of metabolism, superoxides (${\text{O}}_{2}^{-}$). Under physiological conditions, reactive oxygen species (ROS) are readily detoxified to hydrogen peroxide (H_2_O_2_) by the antioxidant enzyme manganese superoxide dismutase (Mn-SOD). Expression of Mn-SOD increases steadily from birth to P60 in the rat brain (42) (Figure 2). The antioxidant enzyme glutathione peroxidase (GPx), further detoxifies H_2_O_2_ to water and oxygen. Unlike Mn-SOD, mitochondrial GPx expression is consistent between the immature and adult brain (42, 43). The cytosolic antioxidant enzymes, copper (Cu), zinc-superoxide dismutase (Zn-SOD) and GPx follow a similar developmental profile to Mn-SOD and mitochondrial GPX. Cu, Zn-SOD increases steadily from P0–P60 while GPx remains consistent through early development (42). The different patterns of expression between these antioxidant enzymes leaves the juvenile brain particularly vulnerable to oxidative stress in the event of TBI.

Additionally, the mitochondria provide important regulatory and protective functions of calcium buffering for the cell, which may influence vulnerabilities of the brain to excitotoxic events. The ability of mitochondria to act as a calcium buffer in the cell under normal conditions allows for increases in respiratory rate and ATP production (44). The difference in mitochondrial calcium influx between the adult and juvenile brain has been a focus of multiple studies. Under normal physiologic conditions (pH 7.0 with ATP), calcium uptake capacity in isolated mitochondria from the adult rat brain was 35–50% higher than in the pediatric (P16–18) brain (29). Interestingly, the same study demonstrated that mitochondrial calcium uptake capacity in the younger brain exceeded that of the adult brain by roughly 36–47% in the absence of ATP. These results suggest an inherent defense mechanism in the immature brain by which they are less vulnerable to calcium excitotoxicity under “extreme conditions,” than the adult brain.

**Figure 3:**
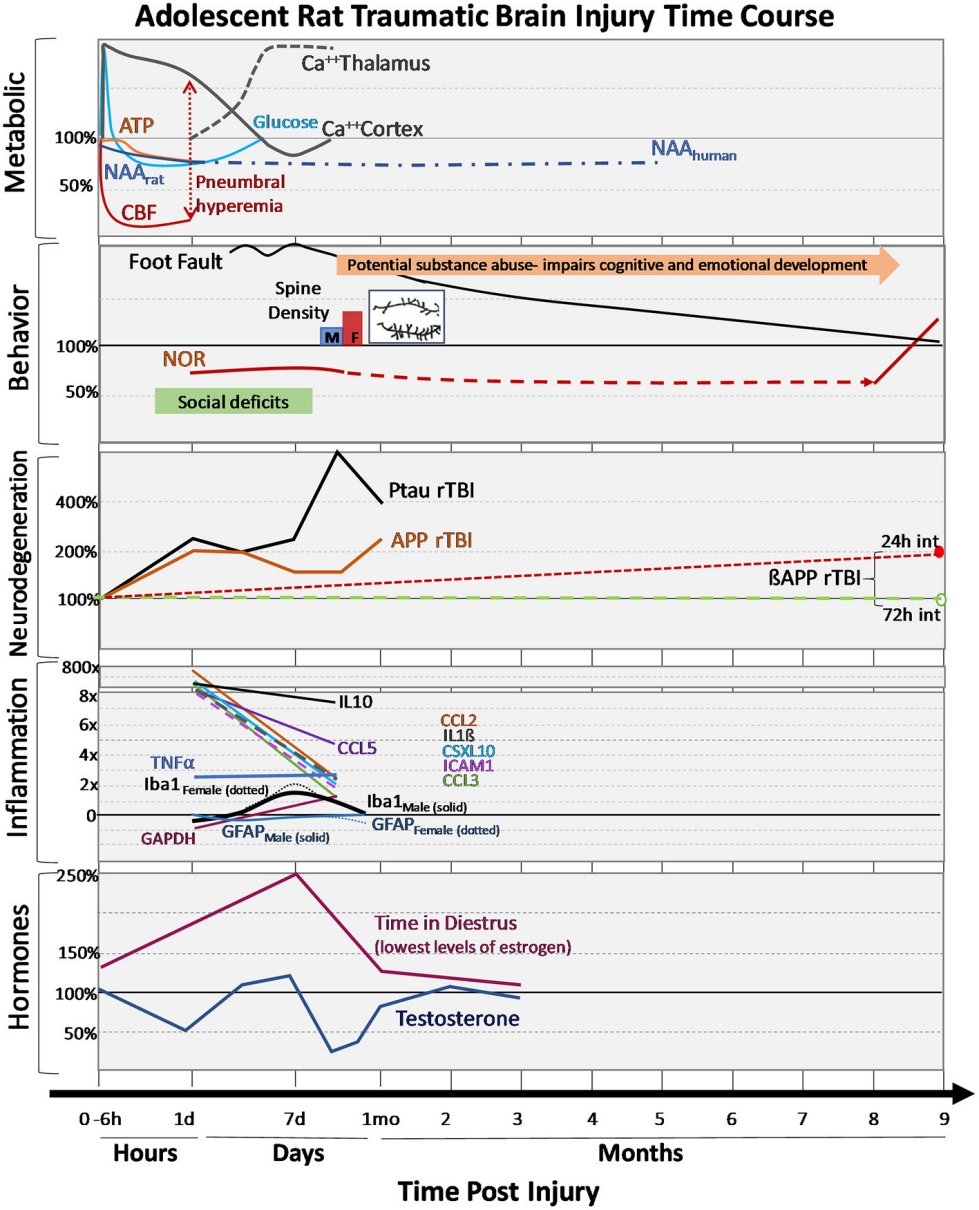
Changes in outcomes after adolescent TBI. Changes in metabolism, behavioral performances, neurodegeneration markers, inflammatory cytokines and hormones after mild-moderate TBI during adolescence are expressed over hours, days, and months post-injury. Changes are expressed as percentages relative to adolescent sham values. **Metabolic**: early dynamic changes in glucose, cerebral blood flow (CBF) with tendency for hyperemia in younger brain, decreases in adenosine triphosphate (ATP) and N-acetylaspartate (NAA), as well as regionally dynamic changes in calcium accumulation as measured by ^45^Ca^++^ accumulation. **Behavior:** Adolescent motor deficits (foot faults) and cognitive (novel object recognition, NOR) deficits recovery can be prolonged recovery, additional stressors like substance abuse can further impair cognitive/emotional recovery, social deficits observed early after injury and the normal process of synaptic pruning (i.e., failure to prune) after injury (spine density: M-males, F-females, with insert showing the post-TBI increase in branching and synapses). **Neurodegeneration:** Following repeat TBI (rTBI), there are acute changes in phosphorylated tau (PTau) and amyloid precursor protein (APP) after injuries. Long-term differences in ßAPP between 24 and 72 h injury intervals are observed in transgenic APP rats. **Inflammation**: Changes in numerous cytokines are shown along with glial fibrillary acidic protein (GFAP, red line), an astrocyte marker in males (solid) and females (dotted), ionized calcium-binding adaptor molecule-1 (Iba-1, black line) a microglia marker in males (solid) and females (dotted). **Hormones:** changes in sex hormones after TBI are shown for males and females.

Given the many developmental changes ongoing in the mitochondria, it is not surprising that age differences in mitochondrial respiratory rates are reported after TBI. While studies have described the patterns of mitochondrial respiration and overall energy metabolism in the adult brain following TBI, there remains limited research in the developing brain. Acute post-injury changes have been reported with a 16 and 35% reduction in complex I and complex II-III activity, respectively (45). These mitochondrial changes likely contribute to early decreases in ATP (30%) and N-acetylaspertate (NAA) (30%) at 24 h after CCI injury in adolescent rats (46) (Figures 1, 3). Other chronic changes in mitochondrial energy production have been reported after weight drop injury and rTBI. Deficits in NAA persist after adolescent rTBI injuries with 60% decrease in NAA/creatine ratio at 7 days post-injury (47). Sex differences were demonstrated in oxygen consumption rates of adolescent rats at 3 weeks post-injury, with females showing greater increases after injury than males (48). One clinical adolescent study supports evidence of long-term suppression of NAA in the anterior corpus callosum at 5 (22%) and 14 (14%) months post moderate/severe TBI (49). While the magnitude and duration of mitochondrial metabolism deficits are not as robust as adults, they still demonstrate the vulnerability of the adolescent brain to energy perturbations after TBI. More research is needed to ascertain the long-term effects of injury on mitochondrial energy metabolism in the developing brain.

There is also limited research on the time course of oxidative stress particularly in the adolescent age group post-TBI. Most of the current research has focused on the immature P17–21 age range of rats, revealing the vulnerabilities in greater accumulation of heavy metals and immature antioxidant systems (50). Among the family of superoxide dismutase, Mn-SOD activity does not reach adult levels until the end of adolescence, and when P17 rats were subjected to CCI injury, they showed a 27% decrease in Mn-SOD relative to age matched controls at 2 weeks post-injury (51). Similar findings were observed with GPx activity, which failed to show increases in P17–21 mice after TBI (43, 52). Clinical observations from infant/children CSF after severe TBI also showed a decrease in antioxidant reserves, ascorbate and glutathione by 5–7 days post-injury (53). More research is needed to understand the time course of oxidative stress and the brain's responses in immature and adolescent age groups, as this is one pathway with a longer maturational trajectory and that contributes to TBI vulnerabilities.

### Inflammation

The immune system consists of innate and adaptive components. Constituents of the peripheral innate immune response include mature granulocytes, macrophages, dendritic cells, and mast cells that function to scan internal processes, discover potential pathogens, and mediate a response to neutralize infections. Activation of the innate immune system initiates the recruitment of cells of the adaptive immune system and the release of cytokines. While the blood brain barrier was initially thought to protect the brain and central nervous system (CNS) from peripheral infiltration of immune cells, new research demonstrates that crosstalk between the CNS and immune cells is crucial for neurodevelopment (54). In the CNS the immune response is mediated primarily by microglia and astrocytes (55). Both cell types have highly specific responses to both injury and infection. When activated, astrocytes and microglia are characterized by phenotypic changes and increased secretion of, or sensitization to, inflammatory cytokines (56). Of the multitude of cytokines and chemokines that can be released, interleukin 1- beta (IL-1β), tumor necrosis factor alpha (TNF-α), interleukin-10 (IL-10), interleukin-6 (IL-6), and interferon gamma (IFNy) are the most prominent and well-studied in current literature.

The immune system has a dual role to both defend against insult and pathogenic infection and to facilitate development of healthy cells. Pro-inflammatory cytokines recruit immune cells and facilitate the continuation of the inflammatory response, while anti-inflammatory cytokines function to turn off the inflammatory response. Pro- and anti-inflammatory messengers fluctuate as a result of homeostatic perturbations, in coordination with normal physiological processes, and change with cerebral maturation [Figure 1 (57)]. In healthy rodent models, age-dependent, stepwise fluctuations from P0 to P30 in cortical IL-1β, IL-1α, IL-6, IFN-γ, and TNFα have been observed (57). These periods of rapidly increasing cytokine concentrations (P0 to P7 and P14 to P30) coincide with developmental milestones, implicating the importance of the chemical messengers in normal neural development. For example, IL-1β has an integral role in neural differentiation and learning and memory, with concentration-dependent function on both neurophysiological processes and neurocognitive performance (58, 59). At 2 weeks of gestation, embryonic rat brain cortices (E14) cultured and exposed to 10 ng/mL IL-1β for 24 h showed normal neuronal differentiation while exposure to higher concentrations of IL-1β (500 ng/mL) had neurotoxic effects (58). Schneider et al. demonstrated that increased IL-1β transcription was associated with long term potentiation in 8 week-old male rats (59). Anti-inflammatory cytokines are implicated in neuronal differentiation and development as well. Astrocyte-derived transforming growth factor beta, an anti-inflammatory cytokine, was shown to regulate neuronal complement component 1q expression, and synaptic pruning during development (60). IL-10 has demonstrated a role in plasticity and in the anti-inflammatory response as well (61). These studies indicate that normal maturation and cognitive development is contingent upon proper balance of pro and anti-inflammatory cytokine concentrations in the CNS (62).

Microglia and astrocyte-derived cytokine and chemokine expression and function in the brain are dependent upon sex as well. Sex differences during development in microglial phenotypes persist into adulthood (Figure 1), as do endogenous cytokine and chemokine expression (63). In developing mice, transcripts for IL-10 and its receptor, IL-10Ra were both significantly increased in females when compared to males at all ages, however this disparity is not reflected by differences in protein expression in adulthood (63). Conversely, significant differences in cortical and hippocampal IL-1β protein concentration persisted through development and into adulthood with females appearing to exhibit greater IL-1β pathway activation (63). A recent study also elucidated sex differences in cytokine expression and elevation following FPI in juvenile rats (64). The average cortical IL-1β concentration was significantly higher in the injured male condition relative to shams 24 h after injury, however this was not observed in the injured female condition (64). Taken together, these findings point to differences in neuroinflammation between sexes throughout development.

The inflammatory response in the brain following TBI is both protective and harmful. In adult animals, the acute inflammatory response following physical insult (e.g., TBI) has been characterized by the quick activation of neuroimmune cells (microglia and macrophages), migration and recruitment of peripheral immune cells, and by pro-inflammatory cytokine and chemokine upregulation (65). This acute inflammatory response exhibits neuroprotective benefits such as the clearance of cellular debris and sealing of the glial limitans (a thin barrier between the brain and spinal cord and the periphery) in an effort to restore cellular homeostasis (66). However, the inflammatory response following TBI is not confined to the site of the injured area, nor does it rapidly dissipate. An exaggerated, diffuse, and/or prolonged inflammatory response is secondary and contributes to a myriad of neurological complications (65).

In juvenile animals, mTBI has been shown to elicit different immunological and inflammatory responses compared to adults. P7 mice have shown an attenuated inflammatory response with an altered time course following TBI (67). Neonatal TBI in mice was accompanied by a localized anti-inflammatory response, as opposed to the diffuse neuroinflammatory response seen in adult TBI (68). Immediately following a CCI injury, these mice showed significant decreases in concentrations of IL-6, IL-1A, IL-10, and IL-1β (68). Additionally, acute increases in TNF-α and IL-1β observed in cortical tissue of adult mice 0–24 h post-mTBI were not observed in P7 mice (66, 67, 69). Following mTBI, P7 mice displayed a consistent >5-fold increase at 6, 14, and 24 h in ipsilateral cortical IL-1β concentrations, while TNF-α decreased from 6 to 24 h. Further, while P30 mice were found to have a significantly elevated immune response compared to sham mice, pro-inflammatory cytokine mRNA upregulation was lower when compared to mice (24 months old) following mTBI (70). Taken together these studies indicate that mTBI elicits different immunological and inflammatory responses in developing and mature brains.

Current research largely points to pro-inflammatory cytokines and immunological cells as causative agents in persistent neurological impairment following TBI, leading to the experimentation of neutralizing cytokines in post-TBI recovery. Bachstetter et al. developed a CNS-penetrant, small molecule experimental therapeutic named MW01-2-151WH (MW151) that restored pro-inflammatory cytokine overproduction toward homeostasis (71). MW151 effectively decreased pro-inflammatory cytokine production and prevented previously observed cognitive decline following TBI. In their study, they found that adult male rats administered MW151 6 h after injury had significantly higher cognitive performance relative to vehicle conditions (71). Similarly, a recent study proposed the NACHT, LRR, and PYD domains-containing protein 3 inflammasome as a potential therapeutic target, citing its effects on cytokine and chemokine production (72). Importantly, these pharmaceutical therapies were administered in adult animals. These treatment options and subsequent fluctuations on inflammatory cytokine and chemokine levels may confer vastly different outcomes in younger animals. Indirect impairment of neurological function and neurophysiological development is a primary concern when treating TBI in pediatric patients. The net benefit of ameliorated neuroinflammation cannot be assessed without an increased understanding of inflammatory and immunological responses following TBI in developing brains.

## Long-Term Outcomes

Overgeneralization of the Kennard Principle has led many to believe that the younger you are when you have a head injury, the better the outcome. While the developing brain shows greater capacity for plasticity, it is also particularly vulnerable. The long-term consequences of early life TBI overlap with the normal brain's maturational trajectory, resulting in more difficult outcome predictions. TBI in the developing brain can slow or shift maturational processes and it is possible for deficits to appear with maturation (growing into the lesion) as well. There are also specific developmental windows during which hormonally regulated changes must occur. TBI can interfere with these processes, causing failure of maturation within these windows. These TBI maturational derailments can also be mitigated by the younger brain's ability for compensation and synaptic plasticity and ultimately impact the long-term outcomes.

### Chronic Cognitive Changes

The impact of TBI on the cognitive development of the young brain has been studied in clinical subjects and preclinical models. In general, when children, 2–15 years old are grouped together, they show significant recovery following mTBI within 6–12 months post-injury in behavioral assessment, cognitive function, and quality of life measures (73, 74). A 2011 study (75), examining children 3–7 and 8–12 years old after mild, moderate and severe brain injuries were evaluated 12 and 30 months after TBI and revealed a susceptibility for severe TBI in young children (age 3–7). Both age groups showed good recovery of cognitive abilities at 12 and 30 months post-injury. With greater injury severity, the young children continued to show cognitive impairments in comparison to older children (75, 76). This pattern has been observed in other studies where younger school aged children with TBI show greater cognitive impairments than adolescent TBI. Children (5–10 years old) and adolescents (11–15 years old) were evaluated at 6, 12, and 24 months post-TBI across all injury severities (77). Adolescent age groups showed greater improvements in math and reading scores over the follow up appointments than children, independent of injury severity. Similarly, Prasad et al. examined the long-term academic effects among children from ages 2 months to 16 years old and reported that students with mild/moderate TBI injuries were only 25% likely to need school services 2 years post-injury compared to those with severe TBI (78). However, 6 years post-injury, the same students were 90% more likely to use school support services. This study also reported that younger ages showed lower functional academic skills than those injured at older ages.

Preclinical studies have not yet replicated the clinical study designs discussed above. One limiting factor when designing developmental research projects is the inability to simultaneously control for age and time after injury. An example of this design limitation is apparent in Rowe et al. (79), where FPI was delivered to P17, P35, and 2, 4, 6 month old rats, but the post-injury assessments were done at 7–10 months of age, not relative to post-injury time points (79). This study demonstrated that the younger rats (P17, P35) showed greater motor and cognitive deficits at 8–10 months of age, than those injured at and older age, who showed greater thigmotaxis in an open field task. Other studies compare developmental age groups or address a specific group. Only a few studies examine long-term cognitive functions post mTBI and rTBI in developing rats. Among P17 rats, mTBI, and rTBI produce mild acute histological and cognitive dysfunction with longer lasting deficits observed at 18–90 days post-injury with increasing number of impacts (80). More moderate-to-severe TBI models at P17 also show long lasting cognitive deficits in Morris water maze, increased anxiety-like behavior, cortical thinning, and corpus callosum damage between 1 and 6 months post-injury (81–84). While acute cognitive changes have been studied in adolescent preclinical injury models, there are no studies examining long-term timepoints.

### Chronic Symptom and Socialization Changes

The CDC estimates that 80% of pediatric TBIs are mild, and that patients display acute affective, cognitive and somatic symptoms that resolve within weeks. However, there are children and adolescents who exhibit persistent symptoms that can interfere with normal developmental trajectories. Starkey et al., studied children 8–15 years old with mTBI by assessing them with the Behavior Assessment System for Children- 2nd edition and Rivermead Post Concussion Questionnaire at baseline and 1, 6, 12, and 24 months post-injury (85). Among the acute symptoms reported, 55% of patients reported headache, and while some recovered, headaches, irritability, frustration, and fatigue were among the persistent symptoms, resolving between 1 and 2 years post-injury. Preclinical models of post-TBI headaches have only emerged in the last few years, addressing the role of rTBI in young adult rats. Tyburski et al., have compared single and repeat closed head injuries in P56 rats, and examined trigeminal allodynia as a surrogate for headache at 3 and 7 days post-injury (86). Findings show that both shorter injury interval and greater number of injuries lowers the sensitivity threshold for the Von Frey fiber both in magnitude and duration (86). RTBI groups showed longer lasting increases in calcitonin gene-related peptide (cGRP) and astrocytosis in the trigeminal nuclei (86). Similar increases in pain sensitivity were observed after weight drop closed head injury in adult male rats for up to 7 days and recovery at 14 days post-injury (87). In this preclinical model, administration of anti-cGRP treatment mitigated pain sensitivity. Ferguson et al., is currently the first examination of allodynia after mTBI in adolescent rats (P35) where rTBI increased sensitivity at 1 day but not at 3, 5, 7 days post-injury (88). Future studies need to address pain sensitivity in both sexes in the developmental age groups.

Findings from a comprehensive literature review on pediatric TBI reports that children are at increased risk of adverse behavioral outcomes after TBI. Children and adolescents with TBI can show impaired communication, socialization, and adaptive behaviors, and with greater injury severity, these impairments can be chronic (89). Children with severe TBI show long term (20 year) deficits in emotional perception compared to those who suffered mild or moderate TBI, and this was associated with decreased volume of corpus callosum and frontal cortex (90). Behavioral issues can be expressed externally (aggression, hyperactivity and disruption) or internally (anxiety, depression, withdrawn). Exacerbation of both types of behaviors have been reported 6 months after mTBI in preschoolers (91). Among adolescents, post-injury chronic behavioral issues can occur 6–12 months post-injury (92). A 2011 Canadian study revealed that adolescents, grades 7 through 12 with a TBI were significantly more likely to attempt suicide, be prescribed medication for anxiety and/or depression, and engage in violent behaviors (93). Behavioral changes have also been addressed post-injury in preclinical models. Social interaction impairments have been observed acutely 7 days post-injury in adolescent rats after mTBI (94). Sham rats were less likely to initiate play with a mTBI rat and the mTBI rats showed greater play avoidance (94). This social learning period is particularly disrupted among females. The chronic consequences are unknown. Shultz et al., examined short-term (24 h) and long-term (8 week) post-injury effects of repeat mTBI on anxiety-like (elevated plus maze) and depressive-like (forced swim test) behaviors. They found t greater the number of rTBI, the greater magnitude of anxiety-like and depressive-like behaviors at 8 weeks post-injury (95). Current scientific findings indicated that TBI during cerebral maturation can impact the normal development of social behaviors, resulting in long-term behavioral changes.

### Adolescent Substance Use and Emotion Perception Post-TBI

Adolescence is a developmental window during which there are normal increases in impulsivity and risk-taking behaviors (96). The late maturation of the prefrontal cortex (PFC), essential for self-control and decision-making, contributes to the adolescent inclination for impulsivity and lack of judgement. This normal developmental trajectory can be further exacerbated by mild to moderate TBI, by delaying executive function network development, which can contribute to poor quality of life in patients overall (97, 98). Additional experimental adolescent tendencies, such as substance abuse are associated with an increased risk for TBI, which highlights the vulnerability of the brain to neurological deficits (99).

Early adolescent TBI can initiate alcohol and/or substance abuse which leads to long-term impaired neuropathological outcomes in adulthood (100, 101). Exposure to alcohol impairs the neurocircuitry of addiction and causes irregular plasticity in reward related learning processes (97). MTBI and neurotoxic effects in adolescent animals confirm the susceptibility of the adolescent brain and the long-term cognitive effects of drinking during adolescence (102, 103). Furthermore, alcohol consumption post-TBI in adolescents affects the ability to return back to work, increases risk for seizures, and inhibits the ability to rehabilitate after a brain injury in later stages. A 2009 longitudinal cohort study, indicated that children who suffered a mTBI before the age of 5 years old were 3.6 times more likely to abuse alcohol during adolescence compared to those with no head injury (104). Adolescents in grades 9–12, with a history of moderate to severe TBI, were twice as likely to binge drink, 2.5 times more likely to smoke cigarettes, almost 3 times more likely to consume non-medical prescription drugs and illegal drugs, compared to those with no history of TBI (105). Similarly, P21 mice who received a mTBI were significantly more likely to increase self-administration of alcohol compared to their adult injured counterparts (103).

Poor impulse control due to TBI and alcohol abuse may have long-term emotional functioning deficits as well. There is limited research on this chronic subject because studying the effects of TBI and alcohol in children/adolescents is difficult due to the matter of the subject and the young age of the patients. However, Dunlop et al. studied adult patients who sustained a moderate to severe TBI and had a history of alcohol use, and found they had emotional and cognitive impairments that persisted more than 6 months after injury (106). This is similar to children, 1–7 years old, who sustained a severe TBI and had difficulty understanding non-verbal emotional cues while experiencing poorer emotional perception compared to non-injured children (107). Adolescents between the ages of 7–17 years old, also had difficulty processing facial emotions and emotional prosody, which allows the understanding of non-verbal aspects of language such as tone of voice, loudness, and pitch, compared to the healthy control group (108). TBI leading to alcohol use and emotional processing deficits may have chronic repercussions for future recovery and future long-term outcomes and needs to be studied in adolescents.

### Plasticity and Pruning

Synaptic density dynamically changes throughout brain maturation and allows the brain to function efficiently and process complex information. Synapse formation can be seen as early as the 23rd week of gestation (109). During the first year of brain development, there is a multitude of synaptic formation between neurons (110). As the brain begins to mature, synapses that are active become strengthened and synapses that are not utilized become pruned. Synaptic density in the parietal cortex of the developing rat has been characterized, showing the increase throughout birth to early adolescence, when pruning begins (111). This process of pruning contributes to healthy brain maturity and brain efficiency perceived in adolescence (112). Almost 50% of synaptic pruning occurs during adolescence, and timing of pruning is dependent on the area of the brain, with higher cognitive function areas pruning through adolescence (110, 113, 114). Research has shown that atypical pruning may contribute to various neurodevelopmental disorders, including schizophrenia, autism, and epilepsy (115).

Given the dynamic nature of synaptic changes, it is not surprising that TBI will induce age specific responses. Introduction of a FPI in the adult rat brain induced a significant decrease in total spine density at 24 h that recovered by 1 week post-injury (116). In contrast to this synaptic withdrawal, injury during adolescence resulted in a different response. Mychasiuk et al. observed abnormal synaptic pruning in post-mTBI during peak maturational growth in P30 rats. Rats were euthanized at P50 and Golgi-Cox analysis of pyramidal neurons in the medial PFC examined dendritic branch order, spine density, neuronal complexity, and dendritic length. Animals with a mTBI had significantly more dendritic branches, significant increase in spine density, neuronal complexity, an dendritic length (117). The consequences of failed or delayed synaptic pruning is unclear. In humans, general research using functional MRI (fMRI) to measure brain activity has found general normalization and recovery in brain function following a childhood TBI (118). There are few longitudinal studies that use fMRI to look at childhood and adolescent brain injury. One longitudinal task-based fMRI study in adolescents aged 15–17 years old with a moderate to severe TBI showed an increase in the left sensorimotor cortex activity during a working memory task, and a limited normalization of acutely increased anterior cingulate cortex activity 12 months post-injury (119). This is similar to the control group's fMRI seen at the initial visit (119). The paucity of research highlights a need to study this area more in order to better understand the long-term pathological effects post-TBI in children and adolescents.

### Hormonal

In current clinical literature there are still gaps to bridge when it comes to the study of sex differences in long-term TBI consequences, especially in the childhood and adolescent population. For this reason, it is important to look at extensive pre-clinical sex-dependent studies in child and adolescent brains since this is a critical time period of brain maturation and growth. Onset of central puberty (i.e., gonadarche) in both humans and rats results in increased production of gonadal hormones. During this phase, re-establishment of the pulsatile release of gonadotropin releasing hormone stimulates production of luteinizing hormone (LH) and follicle-stimulating hormone (FSH) from the anterior pituitary gland that then work on terminal organs, i.e., the testes and ovaries, to produce sex hormones (120, 121). Onset of sexual behaviors begins to emerge shortly after pubertal increases in sex hormones. Increases of sex hormones lead to both the appearance of secondary sex characteristics and increased linear growth (121). Puberty is a sensitive period for gonadal steroids to organize the brain within adolescence (122). Exposure to gonadal sex steroid hormones can be activational or organizational (123–125). Activational exposure usually relates to exposure to a hormone at specific times, which influences behavior. This is usually seen in adulthood and is transient. Organizational effects are those where gonadal sex steroid hormones result in sexually dimorphic development of the brain. In contrast, these structural changes are permanent, exist beyond the exposure to the hormone, and lay the groundwork for activational responses to occur. Prepubertal castration of male rats resulted in reduction of cells within the hypothalamus and amygdala (126). This has also shown to result in impairment in adult sexual behaviors that are not rescued by supplementation with testosterone in adulthood (126). Exposure to elevated amounts of testosterone during puberty increased axon spine density in the amygdala and hippocampus (127). Modulation of brain areas by testosterone has also been observed in human males (122) and has been shown to have effects on later adult social and reproductive behaviors in both humans and animal models (124, 128–132). This suggests a discrete window of development whereby altered sex hormones will result in permanent changes in both brain development and adult behavior.

Pituitary dysfunction has long been recognized as a consequence of TBI, although it was initially considered a rare event. Over the past decade however, a large body of evidence has shown that prevalence of hypopituitarism following TBI is more common than previously thought in both adults and children. Many of the symptoms of hypopituitarism, including disruption of cognitive (memory and decision making), affective (depression and anxiety), and somatic (sexual dysfunction, sleep disorders, increased body mass index) abilities overlap with those reported with symptoms post-TBI. Pituitary dysfunction is not often thought of as a cause of these symptoms until deficits become severe. In adult studies, prevalence of hypopituitarism is present in 23–69% of patients with TBI (133). Variation in reported rates may be due to differences in inclusion criteria and diagnostic methods (e.g., baseline testing vs. provocative testing). The growth hormone and gonadal axes are the most commonly affected (134, 135). Most cases appear to begin to resolve within 1 year, while others worsen between 1 and 3 years (133). It is still unknown how hypopituitarism may contribute to morbidity and mortality following TBI. Pediatric pituitary dysfunction following TBI is poorly understood compared to adults. In the literature there are few studies that observe development of pituitary dysfunction and none address long-term consequences. In the prospective and retrospective studies available, the pediatric population shows an incidence rate of pituitary dysfunction in 16–61% of patients, 1–5 years after injury (136). Cases of pediatric hypopituitarism appear to be divided into two groups: either patients who present with acute dysfunction that resolves within 1 year, or those that have chronic dysfunction still present beyond 1 year post-injury (136), independent of injury severity. It is not clear is whether those with acute deficiencies continue to have chronic deficiencies or whether those with chronic deficiencies develop them over time. However, pediatric TBI are 3 times more likely to have a central endocrine diagnosis compared to the uninjured population (137). Despite the necessity of proper pituitary function for normal physical and brain development, hypopituitarism continues to go unrecognized and untreated in children following TBI. Disruption of normal pituitary function during critical periods of development has the potential to cause long-term cognitive and behavioral deficits.

Causation between isolated hormone deficiencies during childhood/adolescence and perturbed adult function has been shown in several syndromes (123, 138–140) and underscores the importance of proper endocrine function during adolescence. Despite growing evidence showing a clear relationship between TBI and hypopituitarism in children, little research specifically address recovery and development. Few studies have long term quality of life (QoL) scores, neuropsychologic testing and development, but in the studies available, all contain pediatric TBI patients that have acute and chronic hormone deficiencies, abnormal development and increased poor QoL (141–146). One study specifically observed changes in sexual health, an underrepresented area of study following TBI, and found teenagers with a TBI had a higher incidence of poor sexual health compared to age-matched peers (147). In addition to sexual health, disruption of sex hormones results in abnormal menstrual cycles (148, 149) and may also effect fertility. Children and adolescents who had a prior TBI were also reported to have a blunted cortisol response to a social stressor, suggesting alteration of the hypothalamic-pituitary-adrenal axis (150). Basic science and understanding of molecular mechanisms in this area are also lacking in pediatrics. While there are several studies that address pituitary dysfunction following TBI (151–157), these studies have only been done in adult rats. Greco et al., utilized an adolescent mild rTBI model to examine hypopituitarism following TBI. This model resulted in persistent growth hormone, insulin-like growth factor 1 and testosterone deficiencies within 1 week and up to 1 or 2 months post-injury, relatively (158, 159). The distinct lack of experimental studies addressing pediatric pituitary dysfunction is apparent.

While adolescent males are more likely to suffer a TBI compared to females (160), female adolescent concussion rates are higher when a similar sport is considered (161). Further, female student athletes take longer to recover post-TBI (162). Compared to males, however, females also tend to recover better post TBI (163). Females and males produce a sex steroid hormone called progesterone which is involved in the menstrual cycle in females. In females it is secreted by the corpus luteum. While both sexes produce this hormone in the brain and adrenal glands, secretion levels are 10–30 times higher in females during the luteal phase of menstruation (164). Research has consistently shown that progesterone is potentially a neuroprotectant and can decrease cerebral edema, enhance remyelination, and reduce neuronal loss (165). In a 1993 study, false pregnant female rats with highest progesterone levels had no significant edema, and adult female rats in proestrus had significantly less cerebral edema post TBI, compared to adult male rats (166). This indicates that gender specific treatments are necessary to improve quality of life and post-injury outcomes.

While data show there is an opportunity for a window of intervention, there are currently no Class I recommendations on when to screen or begin hormone replacement following TBI in children and adolescents. As such, the literature is divided. In regards to screening, viewpoints range from the incidence of hypopituitarism is so low that it does not warrant any screening (142, 167), screening should only occur if there are obvious delays in growth and/or altered pubertal status (168, 169), and baseline screening for moderate to severely injured patients at 3, 6, and 12 months post-injury (136, 141, 170, 171). One caveat of the current views is the exclusion of screening for those with mTBI; 80% of all TBI are mild. Furthermore, there is no correlation between injury severity and incidence of hypopituitarism nor is there positive findings on CT or MRI scans (172). Another caveat is that not all children with hormonal deficiencies (specifically GH and gonadal hormones) present with delays in growth and pubertal stage advancement. There are currently no predictive measures to determine which pediatric patient is at greater risk for developing hypopituitarism, although one study suggests symptoms of gonadal dysfunction may be the best indicator (149). Also, there are often no specific symptoms, which complicates diagnosis. Often children complain of non-specific symptoms including fatigue, weakness, headaches, visual disturbances, sleep disturbances, impaired executive function and confusion; albeit reported at a higher incidence than adults (173). In addition to being common post-TBI symptoms, these are also common symptoms reported during adolescence caused by normal ongoing neurologic and physiologic changes.

The effects of hormone replacement therapy on morbidity following TBI in children and adolescents are also under-addressed and under-investigated. One of the most important questions to be answered is when to begin hormone replacement therapy and what is the therapeutic window. In most cases hypopituitarism self-resolves within 1 year and has been used as a rationale to dismiss hormone replacement therapy. Because adolescence is a developmental window, it is critical that guidelines be developed around when to screen for pituitary dysfunction and to define the therapeutic window for hormone replacement to maximize the chance of typical neural development and prevent long-term changes in adult behaviors.

### Inflammation

Treatment for TBI is difficult due to the heterogeneity and complexity of events that follow. Primary injury occurs at the time of injury and causes direct damage to tissue. Secondary injury occurs minutes to months later and includes excitotoxicity caused by increased glutamate, free radical generation, and neuroinflammation (174–176). Typically, the immune response is balanced, whereby it is turned on by environmental toxins or injury and is turned off by anti-inflammatory agents after the stimulus is neutralized. In some cases, however, the immune system gets stuck in high gear, known as chronic inflammation. In the brain, glial cells have an extended activation and maintain a pro-inflammatory response for a longer period of time compared to the periphery (177). Following TBI in P21 rats, one study found elevated leukocytes for extended periods, up to 2 weeks post-injury, contrasting with adults where inflammation subsided after 7 days (178, 179). Similarly, following mild rTBI in adolescent human brain endothelial cells, pro-inflammatory cytokines, specifically IL-1β and IL-18, were elevated 2 weeks following injury (180). Another study of adolescent mice given a moderate TBI found pro-inflammatory cytokine expression 2- to-7-fold higher compared to uninjured controls 2 weeks post-injury (181). In the same study, activated astrocytes and microglia were also elevated following both mild and moderate TBI 30 days post-injury demonstrating chronic inflammation. Clinically, some TBI patients have shown chronic neuroinflammation and microglia activation lasting for >17 years following a single injury (182, 183).

Astrocytes and microglia are necessary for the maintenance of the BBB to promote tight associations between cells (184–186). Brain injury or stress, stimulates inflammation and reactive gliosis, causing a disruption and opening of the BBB (184, 186). Specifically, the release of pro-inflammatory cytokines, like IL-1β, can lead to long term permeability in the BBB through structural changes such as reductions in claudin-5 and degradation of basal laminin (187–189). The degradation of the BBB allows for peripheral immune cells to enter the brain to activate microglia and propagate the inflammatory response.

Inappropriate inflammation over a long period of time can lead to tissue damage and subsequent neurodegenerative disease (ADHD, Parkinson's disease, Alzheimer's disease), cognitive deficits, and mood disorders (anxiety and depression). This is particularly true of subcortical regions including the thalamus, putamen, and occipital cortices and has led to white matter damage and reduction in corpus callosum thickness in 28% of the TBI population (182, 183). Long-term inflammation in these studies and others has been associated with more severe cognitive impairments in adulthood. Chronic elevations in IL-1β have been linked to behavioral dysfunction in adolescent mice following a repeat mTBI (180). Further, immune mediators from the periphery can access the brain to activate microglia, which release glutamate and stimulate NMDA receptors, and downregulate dopamine neurotransmission which can lead to depression symptoms (189). Activation of microglia during development can also alter important homeostatic functions, like synaptic pruning, and may lead to cognitive deficits like learning and memory disabilities, as well as mood and sleep disorders (179, 190). In some cases, primary and secondary inflammation of the CNS can lead to childhood inflammatory brain disease like CNS vasculitis or primary angitis of the CNS (PACNS), an acquired neurological deficit in children (191, 192). PACNS is an immune-mediated inflammatory process directed toward blood vessels and can cause seizures, headache, and cognitive decline (192). The same symptoms of seizure, headache, and cognitive decline are often reported following TBI and may also relate to the inflammatory process that occurs following insult. Further, TBI in adolescence, and neuroinflammation in the cortex and mesolimbic track, may promote use of illicit drugs of abuse. One study of adolescent mice has shown acute and chronic inflammation in the nucleus accumbens and ventral tegmental area, regions of the reward circuit, that coincided with a preference for a cocaine-paired environment (181).

Oxidative stress that occurs following injury is greater in pediatrics as well and leads to greater secondary neuroinflammation (53, 179). A study of children 2–16 years old with severe TBI found enhanced oxidative stress, increased free radical production, and reduced antioxidant reserve, different from what has been seen in adults (53). The consequences of this can be beneficial in the pediatric brain, however. One study showed that in rats with a TBI, younger populations (P12 and P21) were resistant to oxidative stress-induced seizures, but not adolescents or adults (P30+) (193). This highlights the heterogeneity of TBI in pediatric populations and emphasizes the dual role inflammation has in response to injury.

## Discussion

Childhood and adolescence is a period of critical ongoing development including changes in synaptogenesis, metabolism, and blood flow. However, many studies focus solely on adults and on short term consequences following a TBI in children and adolescents. Adolescence sees the re-establishment of the pulsatile release of sex hormones. These changes create windows of vulnerability in the pediatric and adolescent population causing detrimental effects from traumatic brain injury. Age differences in acute pathophysiology during cerebral maturation may contribute to long-term pathophysiological consequences. Response to brain injury in the younger population is different from adults. Specifically, the pro-inflammatory response is exaggerated and lasts longer than adults, and metabolic dysfunction, including calcium influx, is less robust in children and adolescents compared to adults. There is also atypical pruning post-injury with some studies showing increased spine density and dendritic branching. The consequence of such changes can lead to neurobehavioral disorders including epilepsy, autism, and even long-term neurodegenerative diseases like Parkinson's and Alzheimer's (194–196). The younger populations, i.e., children and adolescents, seems to be especially vulnerable to social interaction deficits, anxiety, depression, and addiction disorders.

Other things to consider are the effects of exercise and sex on the pediatric and adolescent population response to TBI. It is true that children, 0–14 years old, and adolescents, 15–18 years old, are at greatest risk for mTBI. For adolescents, sports related injuries are the leading cause of single and repeat brain injury. As such, the observed rates for TBI, particularly mild, are most likely lower than the actual occurrence as many mTBI and rTBI go unreported due to the lack of understanding concussion risk factors. Exercise in adolescents have shown positive cognitive, behavioral, and immunological changes which can influence response to injury. Compared to sedentary rodents, adolescent rodents given pre-injury exercise exposure have shown resistance to memory impairment and motor deficits (88, 197, 198). Sex effects have also been observed. While males are more prone to mTBI, risk is increased in females given an equivalent sport, and females take longer to recover following injury. Females and males complain of different symptoms following TBI as well, with females complaining more of headaches and social impairments, while males more often complain of balance and cognitive impairment. Females also show lower cytokine elevation post-injury compared to males, and females may also recover better than males given the production of progesterone.

Despite the given differences observed following injury in the pediatric and adolescent brain, there are few studies that focus on these populations. In particular, more research is needed on the long-term effects following brain injury, particularly in pituitary and hormonal dysfunction, neuroinflammation, and metabolic disruption. Additionally, markers of degeneration such as phosphorylated tau and beta amyloid need to be monitored following single and repeat brain injury in the younger populations. Given that adolescence is a period whereby athletes are beginning their career, it is important to understand lifelong consequences of cumulative TBIs and help improve the overall quality of life of patients who have suffered a TBI.

## Author Contributions

MP assembled the initial manuscript draft and created the figures. All authors wrote sections of the review manuscript, contributed to revisions of the manuscript, and read and approved the final submitted version.

## Conflict of Interest

The authors declare that the research was conducted in the absence of any commercial or financial relationships that could be construed as a potential conflict of interest.
